# Validation of a health screening questionnaire for primary care using Rasch models

**DOI:** 10.1186/s41687-019-0104-7

**Published:** 2019-02-15

**Authors:** Jonathan David Comins, Kirsten Schierup Freund, Karl Bang Christensen, Jørgen Lous, John Brodersen

**Affiliations:** 1 Centre of Research and Education in General Practice, University of Copenhagen, Copenhagen, Denmark; 2 Department of Rheumatology, Zealand University Hospital, Køge, Denmark; 3 Zealand Region Primary Health Care Research Unit, Sorø, Denmark; 4 Department of Public Health, Section of Biostatistics, University of Copenhagen, Copenhagen, Denmark; 5 Research Unit of General Practice, Institute of Public Health, University of Southern Denmark, Odense, Denmark; 6 Department of Orthopedic Surgery and Sports Traumatology, Bispebjerg Hospital, Copenhagen, Denmark

**Keywords:** Primary care practice, Preventive health checks, Consultation process, Clinical interventions, Screening questionnaires, Psychometric properties

## Abstract

**Background:**

Health inequality is on the rise due to various social and individual factors. While preventive health checks (PHC) aim to counteract health inequality, there is robust evidence against the use of PHC in general practice. It is unknown which factors can identify persons who will benefit from preventive interventions that are more beneficial than harmful. Hence, valid screening instruments are needed.

**Methods:**

The aim of this study was to assess the psychometric properties of a screening questionnaire (SQ-33), which targets vulnerable persons in primary care practice who can benefit from preventive consultations. Survey data were acquired from 20 primary care clinical practices in the Northern Region of Jutland, Denmark. Respondents were 2056 persons between 20 and 44 years old who, for any reason, consulted their family doctor. The psychometric properties of the SQ-33 were assessed using Rasch item response modelling. Follow-up analysis was performed on a subsample of 364 persons one year subsequent to initial inclusion, in order to assess responsiveness and predictive validity using a general health anchor item.

**Results:**

Twenty-three of the SQ-33 items in four subscales fit a Graphical loglinear Rasch model (GLLRM) at baseline and follow-up, thus confirming the scaling properties. The modified 23-item version (HSQ-23) revealed superior responsiveness and predictive validity compared with the SQ-33.

**Conclusions:**

The Health Screening Questionnaire (HSQ-23) was shown to possess adequate psychometric properties and responsiveness and can thus be used as an outcome measure in preventive intervention studies. Future study should address whether the HSQ-23 successfully identifies patients who will benefit from PHC consultations.

## Background

Preventive health checks (PHC) has been a controversial topic for at least a decade [1, 2]. There is presently substantial evidence against the use of PHC questionnaires used for screening in primary care medicine [1]. Screening programs can be justifiably implemented so long as the instrument is capable of identifying persons who will benefit from some preventive intervention. However, benefits of screening must always outweigh harms, for example due to unnecessary interventions or overdiagnosis [3–5]. In Denmark, health inequality is on the rise [6], which can be attributed to various social and individual factors [6]. Numerous screening strategies and selection criteria have been applied to identify persons at risk of developing life-threatening or functionally debilitating chronic diseases [7–9]. These strategies include stratifying the general population by age, gender, job-type, financial and sociodemographic factors, as well as specific diagnoses. Nevertheless, while screening instruments should identify persons at risk who can benefit from a preventative intervention, they must not increase the risk of harm by introducing unnecessary diagnoses and treatments [3]. In addition, the instrument must possess acceptable diagnostic test accuracy and consist of meaningful indicators for the target population in order to enhance self-efficacy.

A previous paper describes the development and implementation of a PHC screening instrument for vulnerable persons, called the Screening Questionnaire (SQ-33) [10]. The SQ-33 was developed to assess factors important to health, disease management, and child development using theories of salutogenesis, hierarchy of needs, and self-evaluated health [10]. For a full description of the development of the original SQ-33 questionnaire, and more context, the reader is referred to Freund and Lous (2012) and Hansen et al. (2014) [11, 12].

The domains of the SQ-33 address aspects of Personal Resources (9 items), Lifestyle (8 items), Family Life (10 items), and Relationship with one’s Children) (6 items). The study revealed that a third of the screened population noted difficulties on at least seven of the 33 items [10], and a parallel study found that a number of SQ-33 items correlated positively with certain social and medical conditions and disease states [13]. The results of a 1-year follow-up survey showed that participants randomly assigned to a package of two follow-up consultations with the GP had fewer social problems and an improved sense of psychological well-being compared with controls, as measured by the SF-12 Mental Health Component subscale [11, 12]. This indicates a beneficial impact on these variables. However, evidence of preventive effects on morbidity and mortality remains to be seen.

The SQ-33 screening instrument is a self-report questionnaire where the categorical responses to each item are numericized and summed to a composite score. Summation of raw item scores into a single index (i.e., a *unidimensional* scale) [14] assumes that each item describes a different aspect of the underlying latent trait [15–17]. The summated score is then used as a measure of the degree to which a person with limited resources is at risk of developing a disease, which in turn can affect a person’s health-related quality-of-life (HRQoL).

Item Response Theory (IRT) models are popular and robust statistical tools for validation of scales used to measure HRQoL. IRT models add interesting features to measurement provided adequate data-model fit [18]. Item analyses using Rasch IRT explore in depth which items belong to a single dimension and how items included in each dimension are interrelated and ordered on a latent trait [19, 20]. Good scales exhibit adequate spread along the dimension of interest and are unaffected by subgroups in the population across sociodemographic factors like gender and age [16, 21]. Such person factor bias is known as differential item functioning (DIF) which can undermine the scale if not addressed [22–25]. Local response dependence (LD) is another source of bias that can result in lack of fit to a Rasch model [26]. LD is seen when items are too highly correlated, as items should only be correlated through the latent variable that is being measured [26]. Loglinear Rasch models permit some level of uniform DIF and LD and yet still yield robust scales [27, 28].

Reliability coefficients such as Cronbach’s alpha are often used to estimate measurement error and scale precision at the group level [29, 30]. However, using them to interpret scores at the level of the individual patient is problematic [31], as well as the use of alpha as an estimate of reliability based on a single survey administration [32]. When the purpose of screening instruments is to identify individuals above or below some predetermined cut point, the use of standard error of measurement (SEM) [33] is crucial in order to assess a respondent’s location on a scale relative to that cut point [31, 34].

At present, rigorously validated self-report screening instruments that target vulnerable persons are not available. Notwithstanding, considerable political emphasis has been placed on developing methods to identify premorbid vulnerable persons in order to create interventions that can prevent health inequity and morbidity, and improve HRQoL [7, 9, 11]. Hence, there is a demand for validated self-report measures that consist of relevant indicators which can discriminate between vulnerable and non-vulnerable persons in primary care settings [35, 36].

## Purpose

The purpose of this study was to apply Rasch IRT models to assess the psychometric properties and criterion validity of the SQ-33 as applied to a sample of persons consulting primary care physicians in the Northern Jutland county of Denmark.

## Material and methods

The data used for this analysis were derived from a previously published study of persons who completed the SQ-33 in 1998–99 in the North Jutland County of Denmark [10]. The study included 2056 persons who paid a visit to their GP for any reason, where 1512 (73%) were women. The age within the sample ranged from 20 to 44 years. Of the sample, 495 persons who experienced seven or more problems on the SQ-33 survey were randomised to a 1-h preventive consultation with their GP (and a 20 min follow-up within three months), or to no preventive health consultation [10]. Of the 495 eligible respondents, 364 persons (74%) completed a 1-year follow-up survey (180 persons from the intervention group and 184 from the control group) [11]. Data from the baseline survey (*n* = 2056) were used for the analysis of the measurement properties of the SQ-33 in order to establish the scaling properties for the full range of subjects.

### Analysis strategy

A graphical loglinear Rasch model (GLLRM) was fitted to the proposed subscales [27]. The item screening procedure described by Kreiner and Christensen (2011) [28] was used to identify subscales with adequate fit. Overall model fit was evaluated using Andersen’s conditional likelihood ratio (CLR) test [37], which assesses measurement invariance across groups defined by total score, gender, and age. Individual item fit was assessed by comparing observed and expected item rest-score correlations [28, 38, 39] and by conditional versions of the infit and outfit item fit statistics [28, 38]. A thorough technical description of the criteria used to determine item fit is presented in Kreiner and Christensen (2011) [28]. Measurement precision was evaluated using standard error of measurement (SEM) for the derived scales [33, 40]. The measurement models resulting from the above described procedures were tested in the 1-year follow-up data and the predictive validity of the original 33-item and the reduced versions were compared. Predictive validity evidence refers to the level of correlation between summated domain scores and the anchor item, which is assessed by studying associations between changes in the domain scores with the changes in an anchor item that evaluates general health. The association was calculated as partial Spearman rank correlations adjusting for baseline values of the anchor item. Change scores were calculated as standardized effect size (ES) (i.e., the difference between baseline and follow-up scores relative to the standard deviation of baseline scores) [41, 42].

In order to facilitate comparison of scores across domains, a linear transformation to a zero to 100 scale was used to report domain scores and standard error of measurement.

GLLRM was performed using the software program DIGRAM [39].

## Results

### Resources

**Fig. 1:**
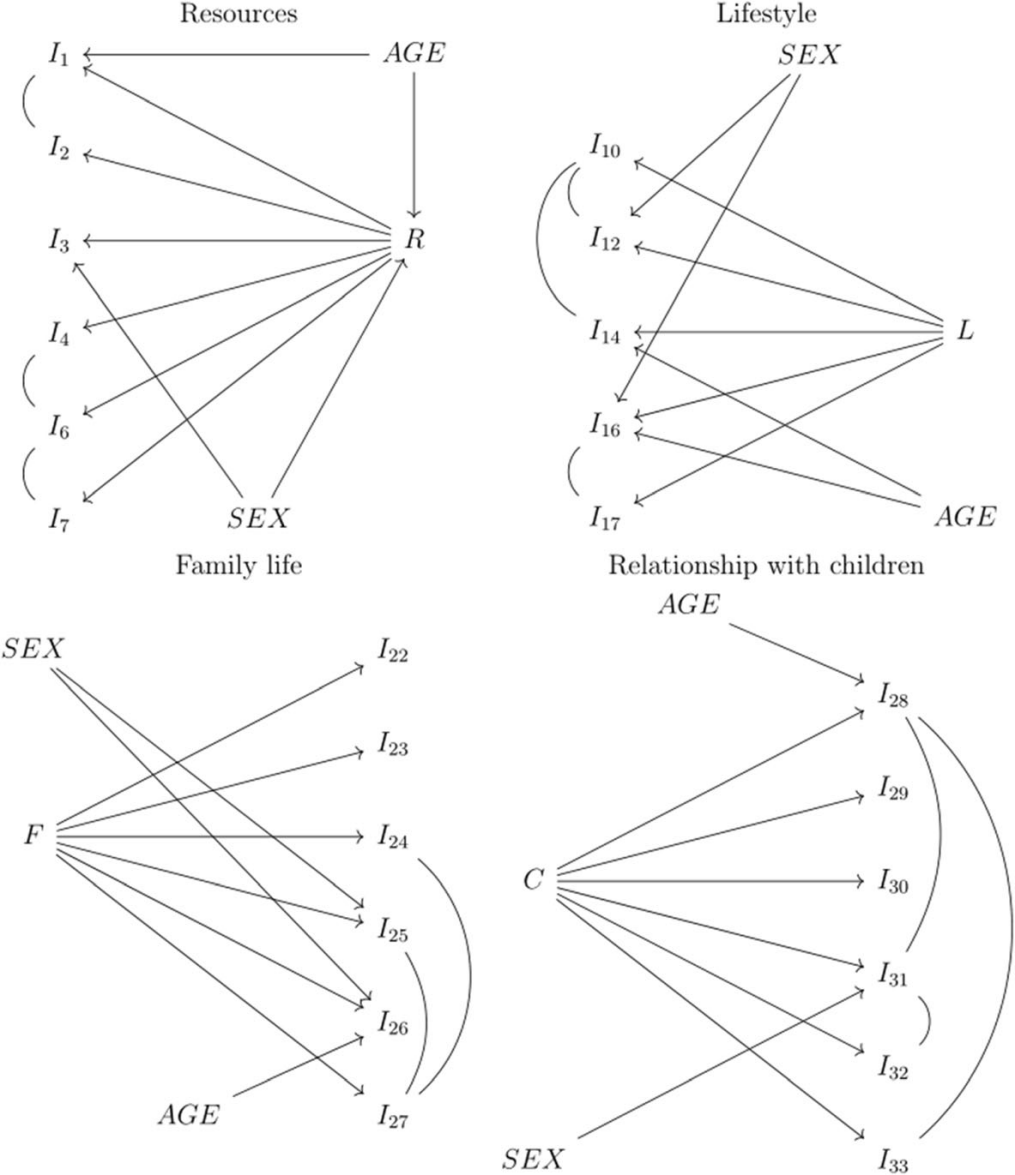
The Graphical loglinear Rasch models

The GLLRM item screening procedure for all nine items (items 1 to 9) indicated massive evidence of positive LD, whereupon no model with satisfactory fit was identified. After deleting items 5, 8, and 9, a relatively parsimonious GLLRM (top left panel in Fig. 1) and adequate overall model fit was identified (Table 1).

**Table 1:** Mean, SD, and over-all model fit evaluated by Andersen’s conditional likelihood ratio (CLR) for each domain

Domain	Mean (SD)	Group	CLR	df	P*
Resources	26.1 (12.6)	Total score	16.8	17	0.4696
		Gender	8.6	13	0.8049
		Age	38.7	28	0.0854
Lifestyle	21.6 (4.8)	Total score	63.1	83	0.9491
		Gender	97.5	75	0.0413
		Age	301.7	256	0.0261
Family life	7.5 (14.9)	Total score	17.3	13	0.1840
		Gender	13.1	9	0.1592
		Age	44.9	32	0.0648
Relationship with children	20.1 (12.2)	Total score	64.4	60	0.3245
		Gender	62.6	52	0.1484
		Age	243.4	192	0.0071

*: No *P*-values significant after adjustment for multiple testing

**Fig. 2:**
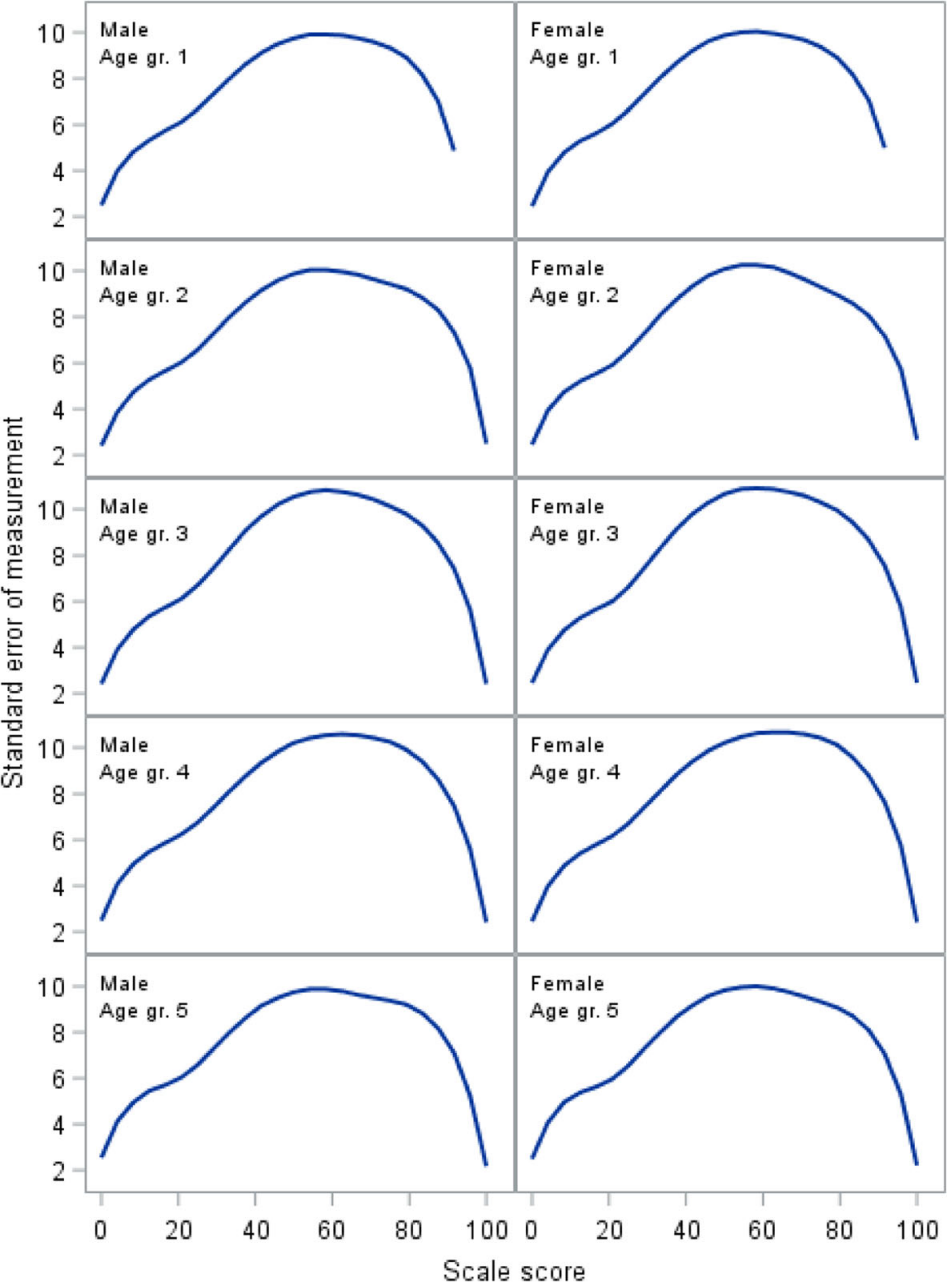
Standard error of measurement for the Resources domain

Individual item fit statistics for the GLLRM analysis are shown in Table 2. Measurement error quantified as the standard error of measurement (SEM) is shown in Fig. 2. The figure illustrates how measurement error for an individual patient can approach 10 points on the zero to 100 scale and differs slightly across gender and age groups.

**Table 2:** Details of the item analysis

	Domain/item	Mean (SD)	Outfit	P	Infit	P	Item-restscore association		
							Obs	Exp	P
	Resources								
1	Sense of general health	2.2 (0.7)	1.06	0.0766	1.05	0.1560	0.58	0.60	0.1246
2	Feel well enough to do what you like to do	1.9 (0.8)	0.98	0.6805	1.02	0.6886	0.63	0.62	0.4612
3	Knowledge about how to improve health and well-being	2.2 (0.7)	0.98	0.5891	0.98	0.5768	0.46	0.43	0.3125
4	Feel appreciated by those you see every day	1.8 (0.8)	1.07	0.0852	1.06	0.1343	0.44	0.49	0.0346
5	Feel appreciated at work	1.8 (0.7)							
6	Ease finding solutions to problems and difficulties in everyday life	2.3 (0.8)	0.95	0.2401	0.99	0.7948	0.61	0.60	0.4777
7	Feel you encounter significant psychological problems in daily life	1.8 (0.9)	0.89	0.0166	0.93	0.0520	0.57	0.54	0.1432
8	Have someone among family you can trust to talk with about possible problems	1.9 (1.2)							
9	Have someone among friends you can trust to talk with about possible problems	2.1 (1.2)							
	Lifestyle								
10	Have felt so stressed that there has been physical discomfort several times a week	1.2 (0.4)	0.96	0.2473	0.96	0.1272	0.24	0.18	0.1341
11	Need time to oneself in daily life	1.4 (0.5)							
12	Have felt the need to reduce consumption of liquor	1.1 (0.3)	1.08	0.3018	1.04	0.4807	0.13	0.22	0.1350
13	Use tobacco on daily basis	1.4 (0.5)							
14	Use pain medications on a daily basis	1.1 (0.3)	1.07	0.4828	1.01	0.9131	0.20	0.26	0.4006
15	Use addictive drugs on a weekly basis	1.0 (0.2)							
16	Vegetables regular part of diet (3 x /week)	1.3 (0.4)	1.02	0.5113	1.02	0.5016	0.22	0.24	0.6124
17	Regular exercise (4 x /week)	1.4 (0.5)	0.98	0.5554	0.99	0.5089	0.23	0.22	0.6631
	Family life								
18	Live alone	1.1 (0.3)							
19	Live alone with one or more children	1.1 (0.3)							
20	Unemployed for more than 6 months in past year	1.1 (0.3)							
21	Unemployed for more than one year in past three years	1.1 (0.3)							
22	Problems with alcohol or drug consumption in the past year for you or your partner	1.1 (0.2)	1.03	0.6188	1.01	0.8274	0.67	0.69	0.5678
23	Problems with alcohol or drug consumption in past year for any of your children	1.0 (0.1)	0.75	0.3803	0.96	0.8565	0.81	0.67	0.1499
24	Significant problems for you in your daily life	1.1 (0.3)	0.97	0.4878	0.95	0.2259	0.82	0.80	0.3455
25	Significant daily problems in your love relationship	1.1 (0.3)	1.03	0.6572	0.98	0.6056	0.82	0.79	0.2520
26	Your child/children have significant daily problems	1.1 (0.2)	1.06	0.4521	1.03	0.6563	0.58	0.66	0.0630
27	Feel secure in daily life	1.1 (0.3)	1.00	0.9739	1.04	0.4086	0.83	0.85	0.4396
	Relationship with children								
28	Assessment of the quality of the relationship with your child/children	1.4 (0.6)	0.87	0.0996	0.92	0.1130	0.50	0.44	0.0973
29	Ability to cope at home or work when your child is sick (e.g., with the flu)	1.8 (0.7)	0.94	0.3145	0.95	0.4091	0.37	0.29	0.0351
30	Actively supporting and improving child’s physical environment (school, transportation, institution, friends)	1.8 (0.9)	1.07	0.1614	1.02	0.6500	0.30	0.31	0.9093
31	Lack energy to put your foot down in the past year regarding your child, even when you feel it is important	2.0 (0.8)	1.00	0.9899	0.98	0.7304	0.42	0.42	0.9847
32	Assessment of own childcare abilities	1.7 (0.7)	1.05	0.2892	1.04	0.4175	0.29	0.34	0.2243
33	Frequent difficulties getting child to eat regularly, and healthy food	2.1 (0.9)	1.07	0.1114	1.05	0.3157	0.33	0.37	0.2923

### Lifestyle

**Fig. 3:**
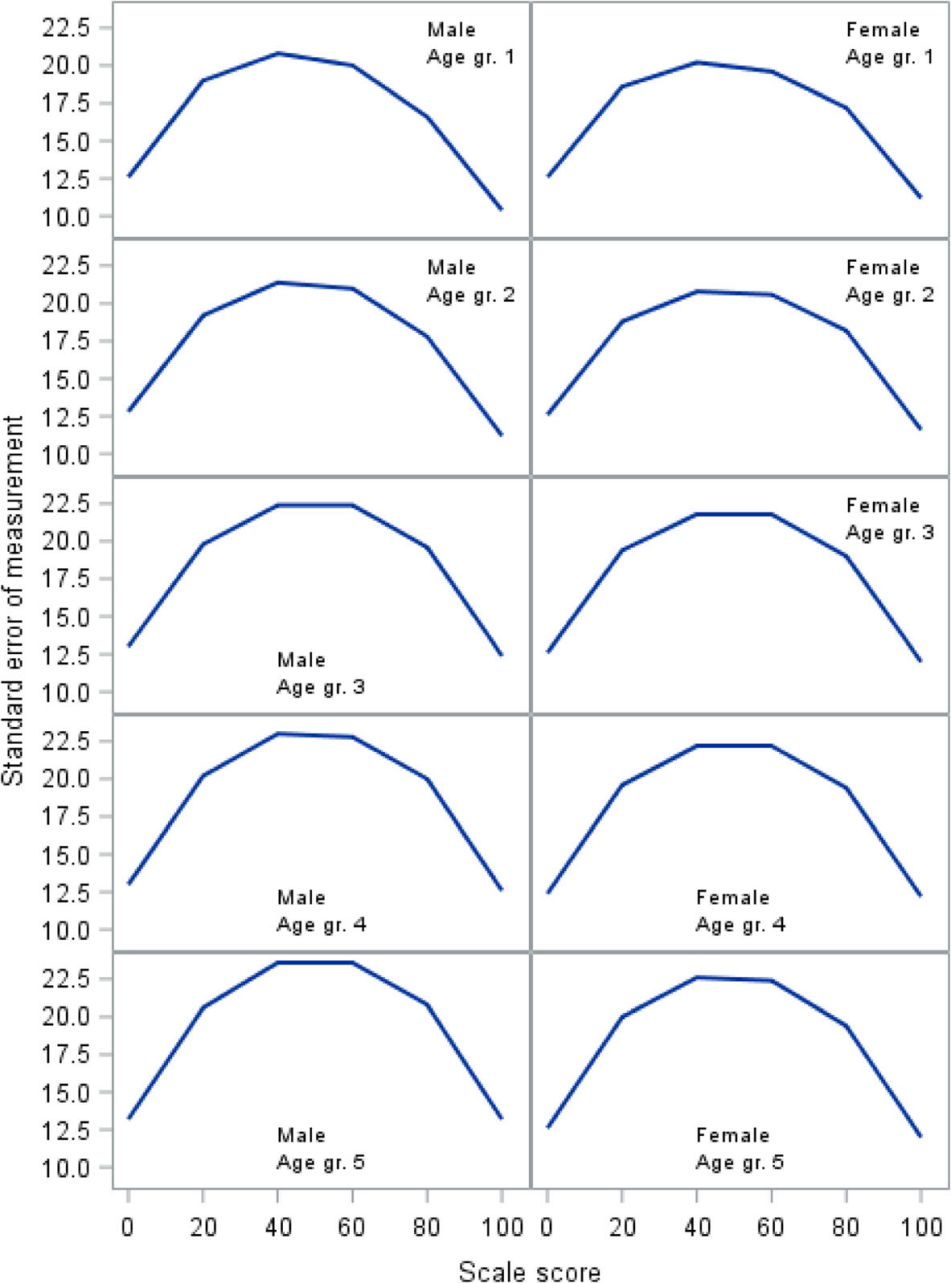
Standard error of measurement for the Lifestyle domain

For the eight lifestyle items, the GLLRM item screening procedure could not identify a model with satisfactory fit for all items. After deletion of items 11, 13, and 15, a GLLRM was identified (Fig. 1, top right panel). Overall model fit was acceptable (Table 1). Individual item fit statistics are shown in the Table 2. The SEM for the GLLRM scale shows that measurement error is extremely large across all age and gender groups (Fig. 3).

### Family life

**Fig. 4:**
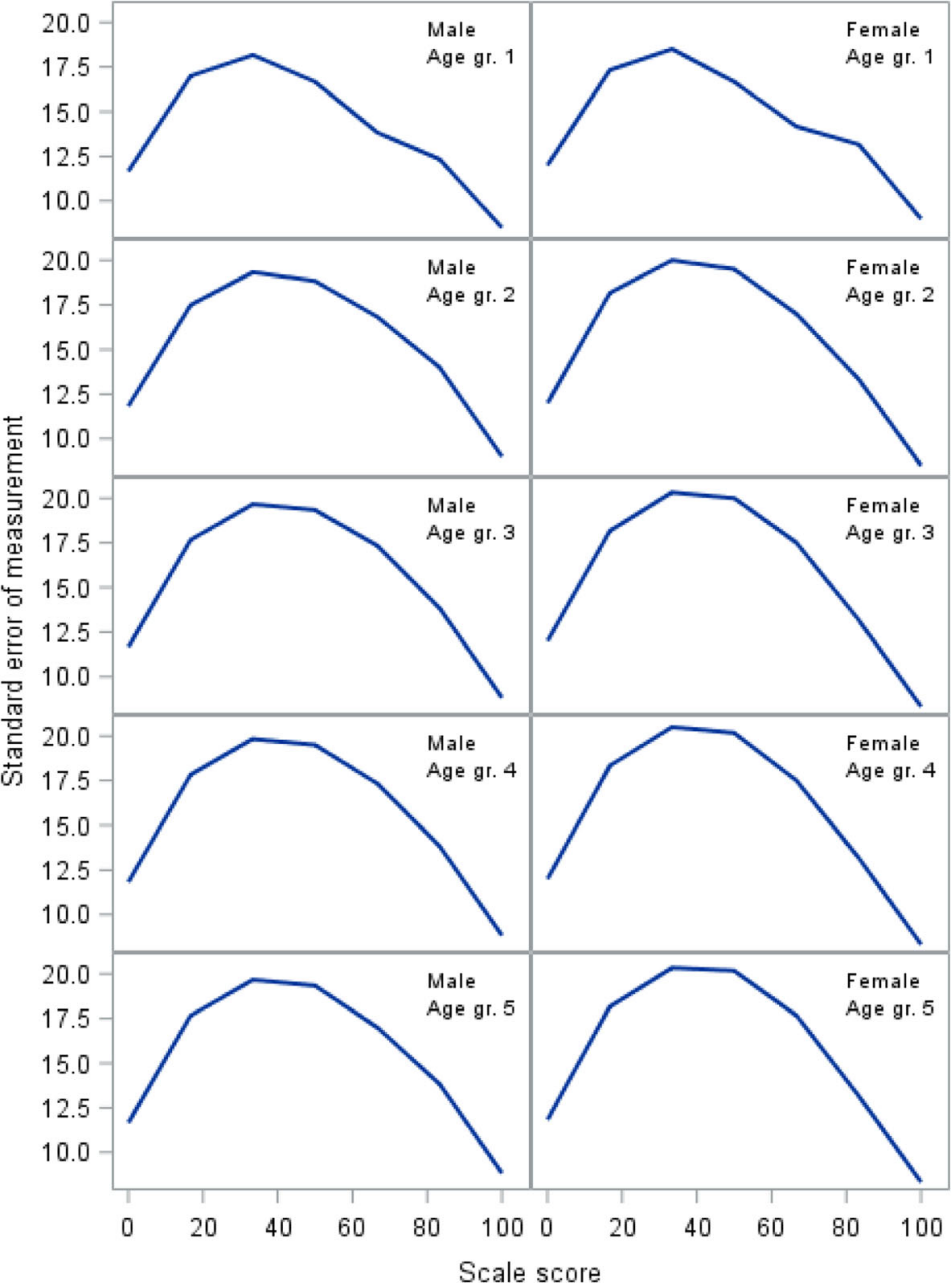
Standard error of measurement for the Family Life domain

The item screening did not identify a model with satisfactory fit for the original 10 item scale. After removal of items 18, 19, 20, and 21, a scale consisting of six items was identified (Fig. 1, bottom left panel). Overall model fit was acceptable (Table 1). Individual item fit statistics are shown in Table 2. The SEM for the GLLRM scale shows that measurement error is very large across all age and gender groups (Fig. 4).

### Relationship with child/children

**Fig. 5:**
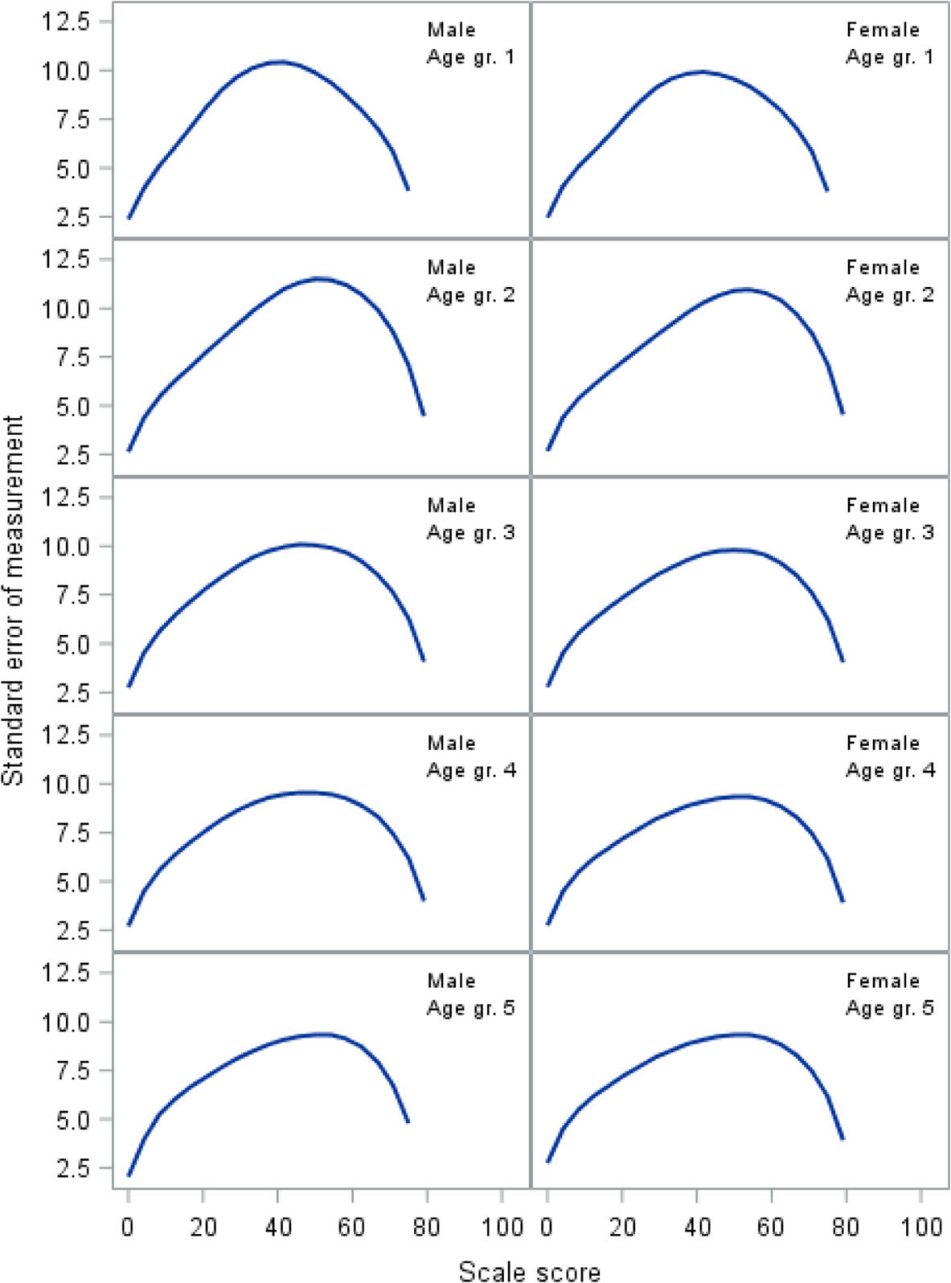
Standard error of measurement for the Relationship with Children domain

The GLLRM item screening procedure indicated that a scale for all six items had satisfactory fit (Table 1). The SEM is shown in Fig. 5 and demonstrates that measurement error is also very large across all age and gender groups.

### Follow-up data

GLLRMs for the four scales identified in the baseline data were confirmed in the follow-up data (results not shown). The standardized effect sizes for the original and for the revised scales are of the same magnitude for all four domains. However, the revised scales consistently show stronger associations with change in the anchor item (see Table 3). Table 3 shows the fit statistics.

**Table 3:** Effect size (ES) and Spearman’s rho for the original and for the revised domain scores

	SQ-33				HSQ-23			
Domain	ES	(95% CI)	rho	(95% CI)	ES	(95% CI)	rho	(95% CI)
Resources	−0.32	(−0.42 to − 0.22)	0.46	(0.38 to 0.54)	− 0.42	(− 0.52 to − 0.32)	0.54	(0.46 to 0.61)
Lifestyle	−1.84	(− 1.93 to − 1.74)	0.00	(− 0.10 to 0.11)	−1.45	(− 1.54 to − 1.35)	0.05	(− 0.06 to 0.15)
Family life	−1.05	(− 1.15 to − 0.96)	0.10	(0.00 to 0.20)	−0.84	(− 0.93 to − 0.74)	0.11	(0.01 to 0.22)
Relationship with children	−0.15	(− 0.26 to − 0.04)	0.19	(0.07 to 0.31)	−0.15	(− 0.26 to − 0.04)	0.19	(0.07 to 0.31)

## Discussion

The most significant finding of this study is that four subscales with twenty-three items satisfy the constraints of GLLRM and can thus be used to measure their underlying constructs in the targeted population. This is encouraging from a measurement perspective. The Resources scale suitably addresses general health, wellbeing, and reflecst a person’s ability to tackle psychological challenges in daily life, which makes sense in terms of content relevance and validity. GLLRM indicated a unidimensional scale, which compensates for some degree of DIF and LD. This implies a reliable, internally consistent, and construct valid measure of the latent variable of Resources for use in population-based studies. DIF by sex for item 3 indicates that men interpret the item differently than women (i.e., a different awareness of how to improve health), which of course is a source of confounding. The fact that men and women respond differentially may warrant DIF equating, as described by Brodersen et al. (2006) [22], in order to quantify the level of discrepancy between genders and compensate in futures studies across groups.

Figures 2, 3, and 4 demonstrate how measurement error distributed across the scale is problematic at the level of an individual respondent, as SEM varies substantially along the scale, reaching peak values in the midrange that are at least twice the magnitude of the low and high ends of the scale. While this is common [31], it has implications for the interpretation of individual scores, as confidence intervals expand with increasing SEM [34]. Hence, depending on an individual’s sum score (i.e., the person’s location on the scale), the uncertainty around the score can differ substantially, which jeopardizes conclusions based on cut points.

Ten of the 33 items did not fit a Rasch model and were removed from the a priori proposed subscales. There can be different reasons for this misfit. For example, two items belonged to the domain of Lifestyle and addressed substance abuse (tobacco and addictive drugs). A potential reason could be that persons with substance abuse are reluctant to respond to questions addressing abuse [43], or the ability to discern the level and influence of the abuse might be distorted by that very abuse or denial [44]. The theme of substance abuse is also captured by items 22 and 23 (alcohol and drug) in the domain of Family Life, so the content does not disappear from the instrument.

It must be noted that items that misfit should be removed from subscales, as they do not contribute to the scaling properties. However, items can always be retained as single items. Thus, information concerning for example a patient’s perceived need to use tobacco on a daily basis can be kept as a single item (and not hidden away inside a scale that possibly measures something else). It is thus the practitioner’s prerogative to use the single item for qualitative assessment.

Other reasons for misfit could stem from local dependence. For example, GLLRM revealed LD between item 1 (sense of general health) and item 2 (feel well enough to do what you like). This makes sense, as both items address general health. Item 3 (knowledge about health) and item 4 (feel appreciated by those you see every day) do not necessarily intuitively address the same topics. It may indicate that cohabitation with family and proximity to friends and colleagues can influence health literacy and self-efficacy.

Poor scaling properties can also be due to the phrasing of the question or the response options [36]. For example, items 18–27 are dichotomous (yes/no). While 6 of these items in fact formed a Rasch scale (items 22–27), dichotomous items may lack nuances that an ordinal response structure capture. Respondents must have adequate response options in order to meaningfully address the item themes. Such qualitative issues can be tackled in face-to-face interviews with the target group in future explorative studies.

A weakness with this study is that follow-up data for just 495 out of the original 2056 persons was obtained (of which 364 participated in follow-up), in that personal identification numbers were registered only for patients with more than seven problems on the SQ-33 in the original collection of data. The rationale behind including persons with 7 or more problems on the SQ-33 for follow-up stems from an a priori assumption by Freund and Lous (2012) that these persons could be classified as ‘vulnerable’ [10, 12, 13]. Excluding the majority of subjects can introduce a bias if the scale is not psychometrically sound. However, the measurement properties of the reduced scales were tested and confirmed in the available follow-up data, and the predictive validity of the revised version was confirmed. Thus, we can conclude that HSQ-23 performs better as a psychometric instrument than the SQ-33 and is responsive to clinical change (as seen by the standardized effect sizes in Table 3).

## Conclusion

Rasch IRT models were used to assess the psychometric properties of the four subscales of the SQ-33 screening questionnaire as applied to persons from the region of Northern Jutland in Denmark. A 23 item version was found to possess adequate psychometric properties and anchor based criterion validation showed responsiveness to clinical change. The revised instrument, the Health Screening Questionnaire 23 (HSQ-23), is appropriate for monitoring the constructs of Resources, Lifestyle, Family Life, and Relationship with children. These scales can be used for outcome assessment in studies of preventive interventions. Whether the scales possess predictive value for specific types of morbidity and mortality is a topic of future investigation.

## Abbreviations

CLR: Conditional likelihood ratio
DIF: Differential item functioning
ES: Effect size
GLLRM: Graphical loglinear Rasch model
GP: General practitioner
HRQoL: Health-related quality-of-life
HSQ-23: Health Screening Questionnaire
IRT: Item response theory
LD: Local dependence
PHC: Preventive health checks
SEM: Standard error of measurement
SQ-33: Screening Questionnaire
